# The Gender-Based Differences in Vulnerability to Ambient Air Pollution and Cerebrovascular Disease Mortality: Evidences Based on 26781 Deaths

**DOI:** 10.5334/gh.849

**Published:** 2020-07-08

**Authors:** Guangcong Liu, Baijun Sun, Lianzheng Yu, Jianping Chen, Bing Han, Yizhuo Li, Jie Chen

**Affiliations:** 1 Department of Occupational and Environmental Health, School of Public Health, China Medical University, Shenyang, CN; 2 Liaoning Key Laboratory of Urban Ecology, Shenyang Academy of Environmental Sciences, Shenyang, CN; 3 Shenyang Center for Disease Control and Prevention, Shenyang, CN; 4 Department of Noncommunicable Chronic Disease Prevention, Liaoning Center for Disease Control and Prevention, Shenyang, CN

**Keywords:** ozone, CEVD, vulnerability, gender differences

## Abstract

The gender-based differences in the vulnerability to ambient air pollution have not been widely explored. This study aimed to investigate vulnerability differences to the short-term effects of PM_2.5_, PM_10_ and O_3_ between cerebrovascular diseases (CEVD) deaths of men and women. The general additive models (GAMs) and distributed lag non-linear models (DLNMs) were adopted, and both single-pollutant and two-pollutant models were performed to analyze the associations between ambient air pollution and daily CEVD deaths. Both models indicated that O_3_ was the most suspicious pollutant that could induce excess CEVD deaths, and women tended to be more vulnerable to O_3_. These results were confirmed by seasonal analysis, in which we also found both genders were more vulnerable to O_3_ in winter. The exposure-response relationships revealed that women were usually more vulnerable to ambient air pollution than men, and the exposure-response curves differed significantly between genders. Our findings suggested that more attention should be paid on the adverse effects of ambient O_3_, and the protection of women CEVD population against air pollution should be emphasized.

## 1 Introduction

The Deaths caused by cerebrovascular diseases (CEVD) account for a large proportion of the population mortality [1]. Recently, the associations between ambient air pollution exposure and CEVD mortality have gained more attention. Some current evidences suggest that short-term exposure to ambient air pollution can increase daily CEVD mortality on the same day or several days after the exposure [2,3,4]. But some studies also indicated no associations between air pollution and CEVD mortality [5,6]. These divergences have not been adequately reviewed, but spatial variations were reported to be a key factor [7]. Spatial variations may include environmental factors, social-economic factors and other domestic characteristics of the study population. During the past few years, the gender-based differences in the vulnerability to ambient air pollution have been given more importance. There are already some evidences implying that the vulnerability to ambient air pollution between the two genders might be considerable different, and women could be more susceptible under most circumstances [8,9]. So far, the age-based differences in susceptibility to air pollution have been frequently reported [10,11,12]. However, few studies about the gender-based differences on deaths caused by CEVD have been published. Moreover, the current environment protection and healthcare policies also pay more attention on the infants, the elderly or people with specific diseases, but seldom take the gender-based vulnerability into consideration. To provide more evidences for sifting susceptible population, further exploration of gender-based vulnerability to ambient air pollution are urgently needed.

On the other hand, low social-economic status are reported to aggravate the vulnerability to air pollution [13]. Therefore, although some relevant studies have been published, those who were carried out under worse air quality and among lower social-economic status population (for example, in developing countries) could yield different results. Therefore, to further explore the effects of short-term ambient air pollution exposure on CEVD mortality, the relevant studies in developing countries with worse air quality are still necessary.

In this study, a time-series analysis was performed. We aimed to: a. explore the associations between short-term ambient PM_2.5_, PM_10_ and O_3_ and daily CEVD mortality; b. detect the potential differences of the gender-based vulnerability to ambient air pollution.

## 2 Materials and Methods

### 2.1 Study area and population

The population of Shenyang, which is the largest city in Northeastern China, were selected. According to the yearbook of Liaoning Province of 2017, the number of Shenyang’s residents was 7.3 million. As a city dedicating to heavy industry, Shenyang was once named as the top 10 heavy polluted cities around the world by the World Bank in 2001. Although the environment has been significantly improved in the past decades, the levels of some air pollutants still kept above the national ambient air quality standard of China (NAAQS). According to the Shenyang Environmental Bulletin, for example, there are 174, 158 and 117 polluted days in Shenyang during 2014, 2015 and 2016, respectively [14].

### 2.2 Data Sources

The daily CEVD (ICD-10, code: I60-I69.9) mortality data during 2014–2017 were obtained from Center of Disease Control (CDC) of Shenyang. The access to the ambient air pollution monitoring data and the meteorological data of Shenyang were presented in detail in a previous study [15]. In brief, daily mean concentration of Particulate matter with diameter <2.5μm (PM_2.5_), Particulate matter with diameter <10μm (PM_10_), Ozone (O_3_) were retrieved from 11 state-controlled environmental air quality automatic monitoring stations through the website of Shenyang Bureau of Ecology and Environment. The overall mean concentration of the 11 stations for each type of pollutant were calculated and used as the general exposure level of the Shenyang population [16,17,18,19]. During 2015 April 4th to 9th, the air quality monitoring data were missing. Therefore, a total of 1455 days’ air quality data (four years total days of 1461 minus 6 days) were available. The source of the meteorological information was from National Oceanic and Atmospheric Administration (NOAA), and was retrieved via the R package ‘worldmet’. Factors such as daily average temperature and daily average relative humidity were extracted.

### 2.3 Statistical Methods

Similar statistical approaches in our previous study were adopted [15]. Briefly, we used a generalized additive model (GAM) with a time series approach, in which daily CEVD deaths were reckoned following a quasi-Poisson distribution. The relationships between daily CEVD deaths and air pollution were considered linear, and both single-pollutant and two-pollutant models were calculated [20,21]. Meteorological factors including relative humidity and daily average temperature were also included.

The models were listed as below (Model 1):

1 \begin{array}{l}
{{\rm{M}}_{\rm{i}}}\ \tilde \ {\rm{quasiPossion(}}{\mu _{\rm{i}}}{\rm{)}}\\
{\rm{Log[}}{\mu _{\rm{i}}}] = {\boldsymbol{\alpha}}  + {{\boldsymbol{\beta}} _1}{{\rm{P}}_{\rm{i}}} + {\rm{ns(RH, d}}{{\rm{f}}_{\rm{1}}}{\rm{)}} + {\rm{ns(T, d}}{{\rm{f}}_{\rm{2}}}{\rm{)}} + {\rm{ns(day}}{{\rm{s}}_{\rm{i}}}{\rm{, d}}{{\rm{f}}_{\rm{3}}}{\rm{)}} + {\rm{Factor(DO}}{{\rm{W}}_{\rm{i}}}{\rm{)}}
\end{array}

Where M_i_ denotes the expected daily CEVD death (including total, men or women) counts of the day i; **α** is the intercept; **β_j_** refers to the corresponding coefficient of ambient air pollutants listed in the models; meteorological factors including daily average temperature (T) and average relative humidity (RH); ns(*X, x*) is the natural spline function; Factor is a categorical datum; DOW_*i*_ is the day of the week on day_*i*_; df_s_ is the degree of freedom of the natural spline function, which is selected from the model with the smallest unbiased risk estimator score; and day_*i*_ is the consecutively numbered day of the study, in which January 1, 2014 is day_1_ and January 1, 2015 is day_366_.

Excess Relative Risk (ERR_10_), which means the excess risks caused by a 10 unit elevation of pollutants, as well as Relative Risk (RR) was used to represent the risks of the ambient air pollutants on the CEVD population. The lag effects during lag0-2, lag0-3, lag0-5, and lag0-7 were explored. To further detect the gender-based differences in the vulnerability to air pollution, we used the Distributed lag nonlinear models (DLNMs) to explore the seasonal exposure-response relationships (during lag0-7) [22]. Considering the exposure-response relationships were not always linear or monotonic, we selected a natural spline function whose dfs were chosen according to the minimum unbiased risk estimator (UBRE) value [23,24,25].

Cross-validations were performed with a leave-1-year-out cross-validation strategy to check the stability of the findings. All statistical processes were performed with the help of the R statistic (version 3.5.1, the comprehensive R Archive Network, http://cran.r-project.org/).

## 3 Results

### 3.1 CEVD Mortality

**Figure 1:**
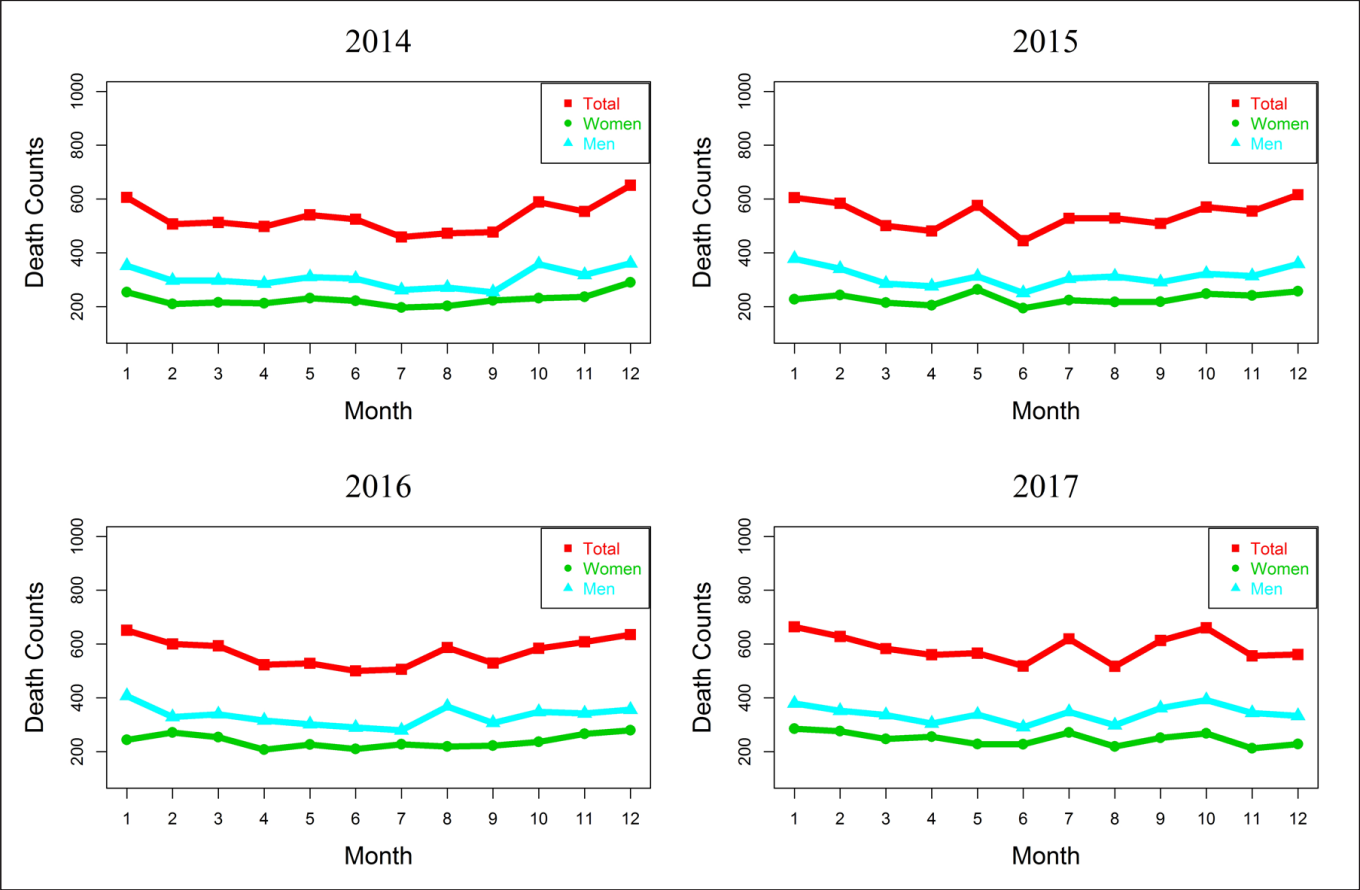
Monthly CEVD deaths during 2014–2017. Generally CEVD deaths of women were more than those of men, and CEVD deaths were more in cold season than in warm season in both men and women.

There were 26,781 CEVD deaths in Shenyang City during 2014–2017, in which the daily deaths of men (57.79%) were significantly more than women (42.21%, P < 0.01). More deaths occurred in cold period (November to February) than in other periods. As to the deaths in each month, CEVD deaths were at peak in January with a 4 year average monthly CEVD deaths of 664, and declined to the minimum of 517 deaths in August. Details about the CEVD deaths in Shenyang during 2014-2017 were presented in Figure 1 and Table 1.

**Table 1:** Descriptives of daily CEVD deaths during 2014–2017.

Year		Total			Men			Women	

	Counts	Daily Mean	SD	Counts	Daily Mean	SD	Counts	Daily Mean	SD

**2014**	6393	17.52	4.62	3670	10.05	3.42	2723	7.46	2.80
**2015**	6499	18.10	4.65	3746	10.43	3.26	2753	7.67	2.89
**2016**	6844	18.70	4.67	3983	10.88	3.36	2861	7.82	3.00
**2017**	7045	19.30	5.41	4078	11.17	4.00	2967	8.13	2.99
**All Time**	26781	18.41	4.89	15477	10.64	3.55	11304	7.77	2.93

### 3.2 Associations between pollutants and CEVD deaths

**Figure 2:**
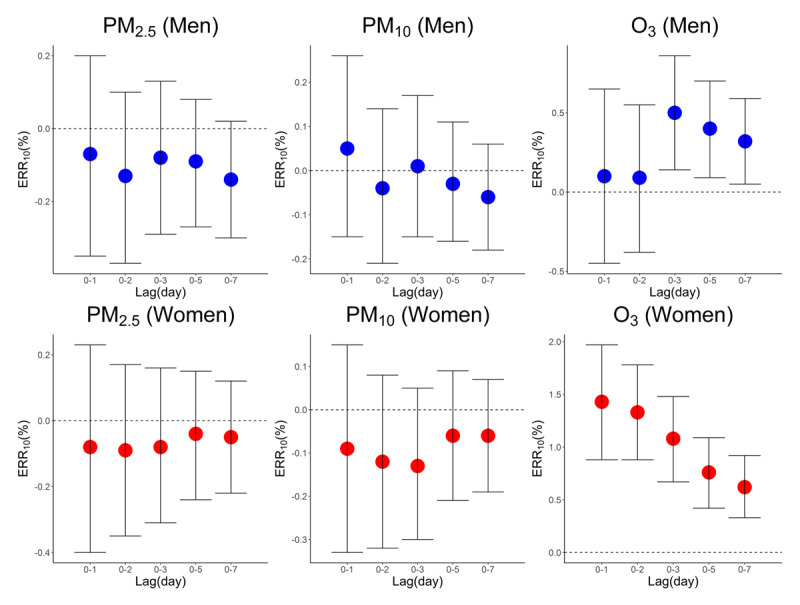
The associations between ambient air pollution and daily CEVD mortality. O_3_ seems to be the most suspicious pollutant that leads to excess CEVD mortality. Generally women with CEVD were more vulnerable to ambient air pollutants.

In the two-pollutant models, we found short-term O_3_ exposure, instead of PM_2.5_ or PM_10_, was positively associated with daily CEVD deaths, implying O_3_ might be the most suspicious air pollutant that lead to the excess CEVD mortality. For example, with the ERR_10_ of total CEVD deaths during lag0-1, lag0-2 and lag0-3 were 1.02% (95%CI 0.63%–1.4%), 0.95% (95%CI 0.62%–1.27%) and 0.74% (95%CI 0.46%–1.03%), respectively. Women tended to be more vulnerable to O_3_ that the ERRs of women were generally larger than those of men. In addition, the ERRs of women decreased with the extension of the lag period, while the ERRs of men increased at lag0-3 and then decreased with time. No positive associations were found between PM_2.5_ or PM_10_ and CEVD deaths. The results of the single-pollutant models varied slightly from those in the two-pollutant models. The association between short-term ambient air pollution exposure and CEVD mortality were shown in Table 2 and Figure 2 (from the two-pollutant model) and Figure S1 (from the single-pollutant model) of supplementary files.

**Table 2:** Cumulative ERR_10_ of each pollutant at different lags (Unit: %).

Lags	Pollutants	Single-pollutant model						Two-pollutant model					

		Men		Women		All		Men		Women		All	

		ERR	Sig	ERR	Sig	ERR	Sig	ERR	Sig	ERR	Sig	ERR	Sig

**lag0-1**	PM_2.5_	–0.07		–0.03		–0.01		–0.07		–0.08		–0.04	
	PM_10_	0.05		–0.06		0.03		0.05		–0.09		0.01	
	O_3_	0.09		1.42	*	1.01	*	0.1		1.43	*	1.02	*
**lag0-2**	PM_2.5_	–0.13		–0.04		–0.06		–0.13		–0.09		–0.1	
	PM_10_	–0.04		–0.1		–0.05		–0.04		–0.12		–0.07	
	O_3_	0.08		1.32	*	0.94	*	0.09		1.33	*	0.95	*
**lag0-3**	PM_2.5_	–0.07		–0.05		–0.06		–0.08		–0.08		–0.09	
	PM_10_	0.02		–0.12		–0.05		0.01		–0.13		–0.06	
	O_3_	0.49	*	1.07	*	0.74	*	0.5	*	1.08	*	0.74	*
**lag0-5**	PM_2.5_	–0.08		–0.02		–0.06		–0.09		–0.04		–0.08	
	PM_10_	–0.02		–0.05		–0.04		–0.03		–0.06		–0.05	
	O_3_	0.39	*	0.75	*	0.54	*	0.4	*	0.76	*	0.55	*
**lag0-7**	PM_2.5_	–0.13		–0.03		–0.09		–0.14		–0.05		–0.1	
	PM_10_	–0.05		–0.05		–0.05		–0.06		–0.06		–0.06	
	O_3_	0.31	*	0.62	*	0.43	*	0.32	*	0.62	*	0.44	*

### 3.3 Seasonal analyses

The Seasonal analyses by two-pollutant model indicated that the CEVD population were more vulnerable to air pollution in cold seasons (winter and spring). In cold seasons, for example, the ERRs of O_3_ among both men and women were mostly significantly above 0. However, we can hardly find significant positive associations in summer or autumn. Some negative associations were found between PMs and CEVD deaths. They might suggest non-linear relationships between short-term PMs exposure and CEVD deaths, since air pollution exposure cannot be a protective factor. Even in the seasonal analyses, the ERRs of women were generally larger than those of men, and this was more obvious in ERRs of O_3_ during winter. The details of seasonal analyses are shown in Table 3.

**Table 3:** Seasonal analyses of the associations between short-term ambient air pollution exposure and CEVD death.

Group	Lags	Pollutant	Spring		Summer		Autumn		Winter	

			ERR	Sig	ERR	Sig	ERR	Sig	ERR	Sig

**Women**	lag0-1	PM_2.5_	–0.1		–0.07		0.05		0.18	
		PM_10_	–0.28		–0.35		0.06		0.2	
		O_3_	1.39	*	0.07		1.51	*	4.12	*
	lag0-2	PM_2.5_	0.19		–0.11		0.03		–0.24	
		PM_10_	–0.16		–0.39		0.04		–0.09	
		O_3_	1.65	*	0.32		0.9		3.2	*
	lag0-3	PM_2.5_	0.06		–0.54		0.05		–0.26	
		PM_10_	–0.18		–0.8		0.05		–0.15	
		O_3_	1.25	*	0.34		0.45		2.78	*
	lag0-5	PM_2.5_	0.2		–0.66		0.13		–0.41	*
		PM_10_	0.11		–0.75		0.13		–0.18	
		O_3_	1.36	*	0.29		–0.2		2.23	*
	lag0-7	PM_2.5_	0.13		–0.72		0.08		–0.25	
		PM_10_	0.02		–0.9	*	0.09		–0.04	
		O_3_	1.37	*	0.33		–0.31		2.44	*
**Men**	lag0-1	PM_2.5_	–0.29		–1.68		0.04		–0.12	
		PM_10_	0.67	*	–0.89		–0.02		0.22	
		O_3_	1.29	*	–0.13		–0.99		1.57	
	lag0-2	PM_2.5_	–0.29		–1.48		–0.06		–0.27	
		PM_10_	0.25		–0.9		–0.1		0.08	
		O_3_	1.24	*	–0.11		–1.04		1.41	
	lag0-3	PM_2.5_	–0.69		–1.41	*	–0.1		–0.12	
		PM_10_	–0.03		–0.93	*	–0.11		0.17	
		O_3_	1.47	*	–0.05		–1.14	*	1.04	
	lag0-5	PM_2.5_	–0.61		–2.01	*	–0.17		0.03	
		PM_10_	–0.02		–1.47	*	–0.17		0.18	
		O_3_	0.99	*	–0.03		–0.8		1.34	*
	lag0-7	PM_2.5_	–0.6		–1.16	*	–0.28	*	0.12	
		PM_10_	–0.02		–1.33	*	–0.27	*	0.23	*
		O_3_	0.54		–0.14		–0.63		1.57	*

**Figure 3:**
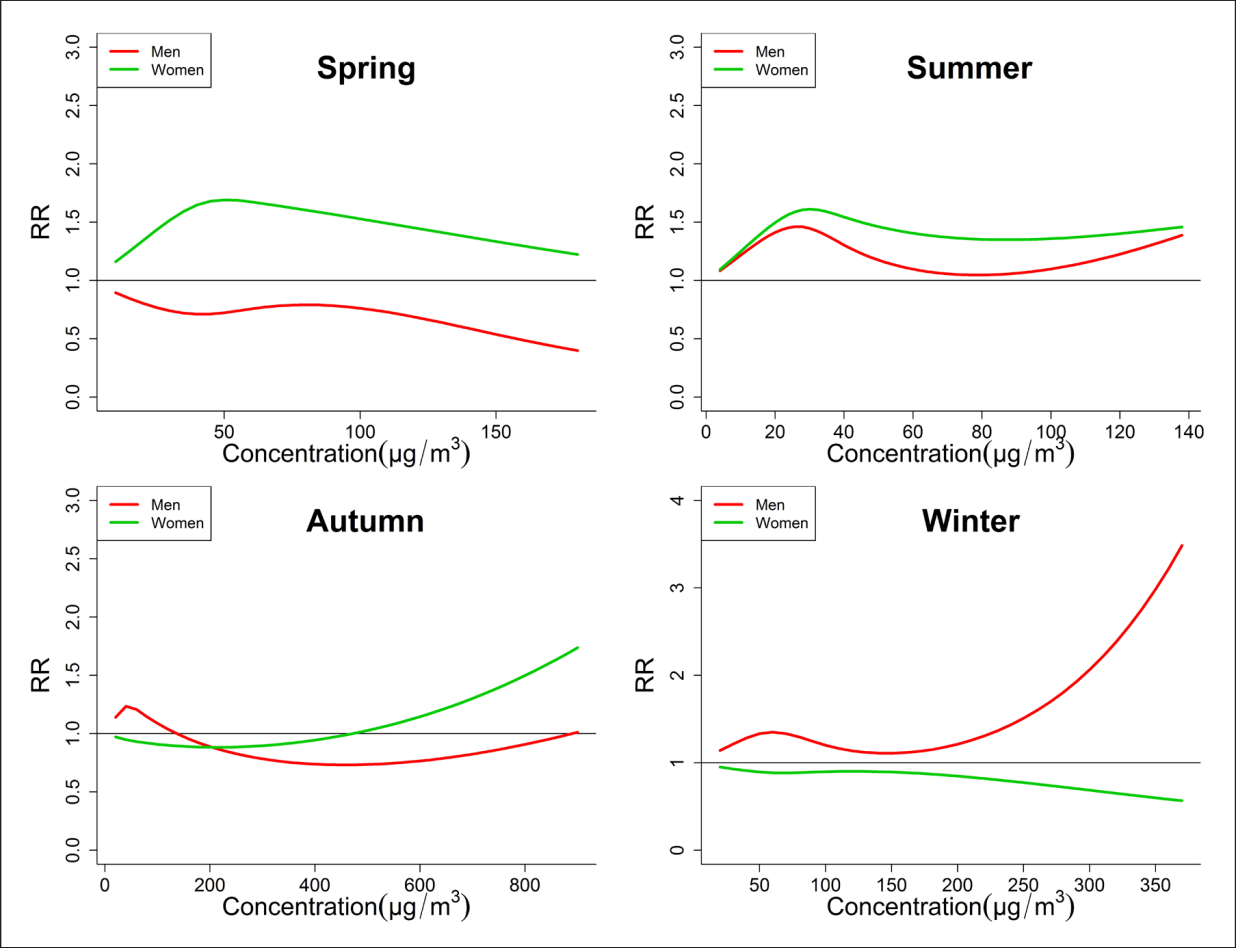
Comparison of Seasonal exposure-response curves of PM_2.5_-CEVD mortality between men and women. Most curves of both men and women tended to be non-linear, and the RRs of women were generally larger than those of men. However, men CEVD mortality were shown to be more vulnerable to PM_2.5_ in winter.

**Figure 4:**
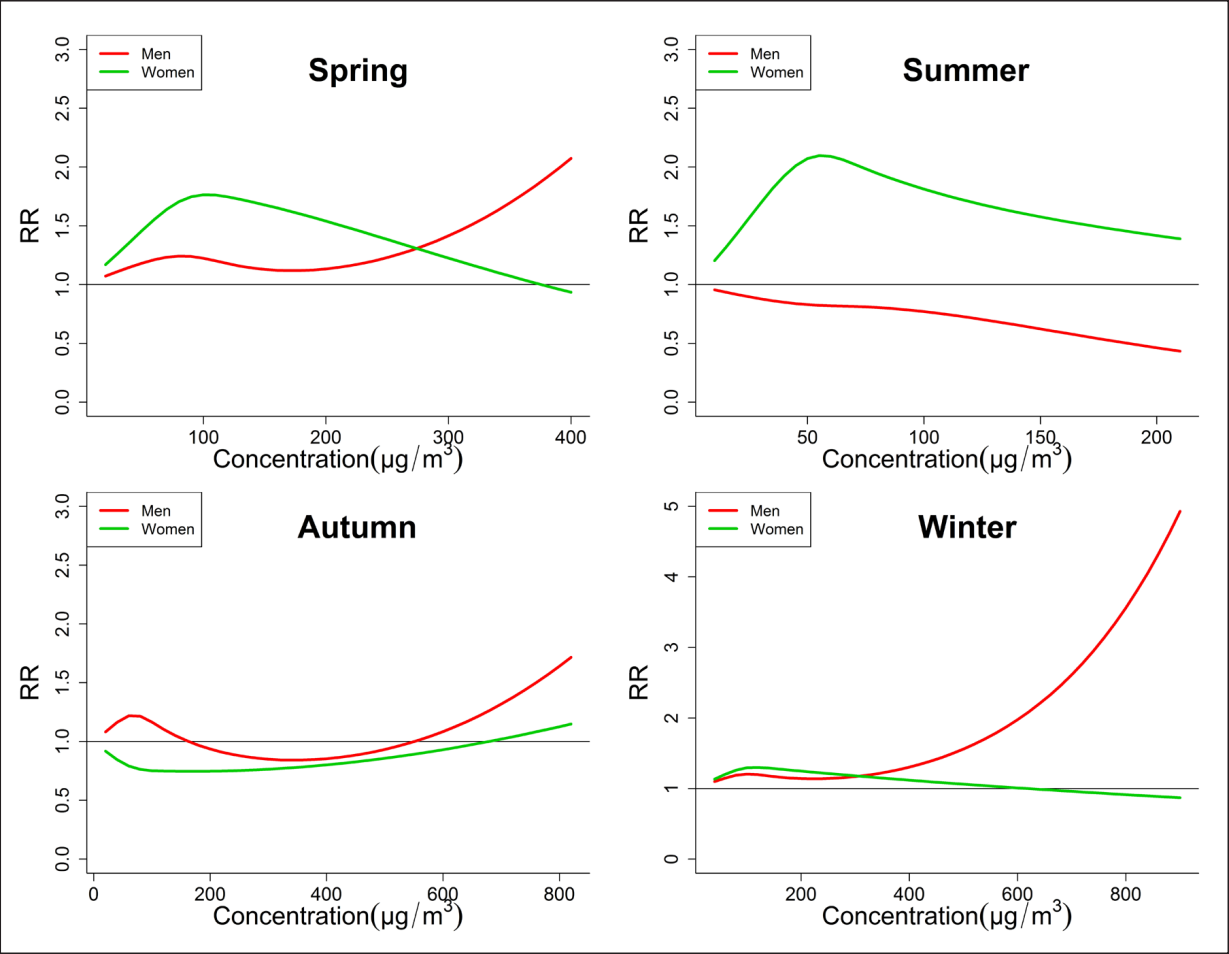
Comparison of Seasonal exposure-response curves of PM_10_-CEVD mortality between men and women. Some curves showed both non-linear and generally increasing or decreasing trend, which may provide a possible explanation for the insignificant associations between PMs and daily CEVD deaths. Generally women were more vulnerable to PM_10_ in spring and summer, but men were more vulnerable in autumn and winter.

**Figure 5:**
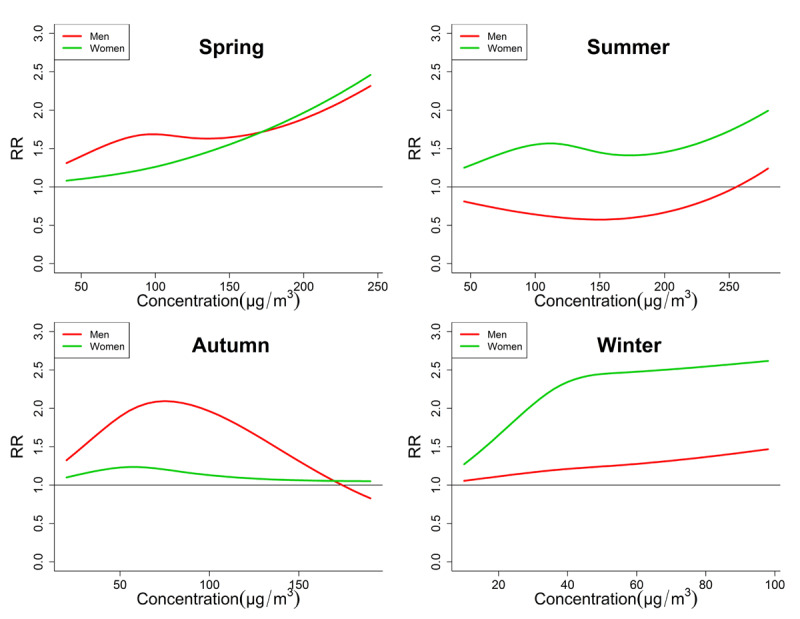
Comparison of Seasonal exposure-response curves of O_3_-CEVD mortality between men and women. Significant differences of gender-based vulnerability were observed. In spring and autumn, men were more vulnerable, but in the other seasons women were more vulnerable.

The seasonal exposure-response relationships of PM_2.5_, PM_10_ and O_3_ by two-pollutant were shown in Figures 3, 4 and 5, respectively. Among these curves, some unignorable non-linear curves confirmed our hypothesis that the exposure-response relationships were not always linear. For example, the curves of O_3_ in autumn (Figure 5) of both genders are obviously not linear, which usually increased first and then decreased. Some curves also increased and then decreased, but they showed a generally decreasing trend, such as the PM_10_ curve of men in spring and winter. This can possibly explain some of the negative associations between PMs and CEVD mortality in the linear models. Another notable result is that the trends of curves sometimes differed significantly between men and women. For example, the O_3_ exposure-response curve of men in autumn was obviously bell-shaped, but the curve of women in autumn showed a slightly bell-shaped trend. And the PM_2.5_ or PM_10_ curves of men in winter generally increased, while those curves of women were generally decreased.

No significant variations were found when using the eliminated data of one-year each time, suggesting good robustness of the results.

## 4 Discussion

In this study, we found limited evidences that PMs could induce increased CEVD mortality when adopting linear relationships. However, O_3_ was shown to associate with increased daily CEVD mortality in both men and women, and women tended to be more vulnerable. These findings implied that O_3_ could be the primary risk pollutant of the excess CEVD deaths, and women were more vulnerable to ambient air pollutants than men.

We found the ERRs of women were larger than those of men in general. This accorded with the previous outcomes among Shanghai, Hong Kong and Japanese population [26,27,28,29]. They are also supported by the mechanism studies indicating that women were more susceptible to oxidative stress (OS) [30]. Bell et al. suggested geographical variation in the magnitude of gender difference, which were larger in the Northeast than in other regions of the US [26]. This is also similar to our study that was performed in the North, since some studies that were performed in middle or southern China indicated no gender differences or men were more vulnerable. However, a recent comprehensive assessment by EPA noted there were not enough evidences to draw a conclusion on this issue [31]. Therefore more relevant studies are still in need.

In addition, we found men might be more vulnerable to PM_2.5_ in winter. Although more papers reported women as the susceptible gender to ambient air pollution, there are also studies indicated that the mortality of men was more sensitive to PM_2.5_ [9,32,33]. These variations of the gender-based vulnerability to PM_2.5_ across the studies may be due to different components of PMs as well as their proportions, since the components vary significantly across regions [34,35]. The haze is also a key risk for circulatory mortality, and most of the haze days in Shenyang occurred in winter [36]. This may also aggravate the adverse effects of short-term air pollution on the CEVD population. As to short-term O_3_ exposure, it has been frequently reported to associate with elevated risk of the increased circulatory mortality. In this study, we further confirmed ambient O_3_ as a fatal risk for CEVD population, as well as the gender-based vulnerability differences. The mechanisms that O_3_ induces excess CEVD mortality are not yet clear, but the influences of O_3_ on circulatory system have been validated by previous studies. One possible path is by inducing hypertension, when ET-1 were generated after short term exposure to O_3_ and then further elevated the blood pressure [37]. And vasoconstriction caused by O_3_ exposure not only induces hypertension, but can also slow the vascular flow, which was also fatal for the CEVD population [38].

Seasonal analyses suggested that both men and women CEVD were more vulnerable to air pollution in cold seasons (winter and spring), and It was more evident in the ERRs of O_3_. Some previous papers reported that O_3_ could elevate the cardiovascular mortality only in warm seasons, but more recent studies (especially those carried out in east Asia) reported more robust associations in cold seasons [29,39,40,41]. In this study, the seasonal variations could largely attribute to the cold climate in Shenyang City. The mean of the daily lowest temperature was –13.5°C in Shenyang’ winter during 2014 to 2017. As the triggers of CEVD deaths, the increased blood pressure and blood viscosity can be induced by the exposure to low ambient temperatures [42]. Several studies also indicated that low ambient temperature can exacerbate the fatal effects of air pollution [36]. Therefore, even if average daily O_3_ concentration were significantly lower than in cold seasons than in warm seasons, the CEVD population of Shenyang could also be more sensitive.

It is interesting that the result of CEVD differed from those of CHD in Liu et al., that CHD mortality was associated with PM_2.5_ while CEVD mortality was associated with O_3_. This may be attributed to the negative correlation between O_3_ and PM_2.5_, and the different triggers of acute onset between CHD and CEVD. First, in this study the ERRs of O_3_ is higher than PMs, indicating that O_3_ induces more risks in the CEVD population of Shenyang. Second, the paths of air pollution-induced acute CHD and acute CEVD may be different. Many studies proved that short-term exposure to either PM_2.5_ or O_3_ would result in acute increase of blood pressure, which is possibly how short-term air pollution exposure increased CEVD mortality [43,44,45]. However, the role of acute increasing blood pressure in inducing the onset of acute CHD was not as strong as in inducing acute CEVD (such as hemorrhagic stroke). Third, the concentration of PM_2.5_ and O_3_ were negatively correlated in Shenyang (shown in supplementary files). In summary, it is possible that in the linear models O_3_ associated with CEVD mortality while PM_2.5_ not.

It is not rare that no associations between PMs and daily CEVD deaths were found in men or women. Several studies failed to prove the short-term PMs exposure as a risk factor that could elevate daily cardiovascular hospital admissions or mortality [46,47]. One main reason for these inconsistencies is that the exposure-response relationships can be non-linear [23,24,25]. Moreover our previous study also indicated that this relationship between ASHD mortality and air pollution seemed to be non-linear [15]. Just as shown in Figures 3, 4 and 5, some exposure-response curves of PMs were bell-shaped, so if they were treated as linear the generally tendency can be decreasing. On the other hand, the inconsistencies may also be attributed to spatial variation; however the potential influences of detailed individual factors remained to be explored.

This study provided the further evidence that women CEVD population were more vulnerable to ambient air pollution, especially more vulnerable to O_3_. It also offered evidence for policy making. The public attention should not only focus on the total air quality, but also on each type of pollutants as well as their susceptible population. In addition, the concentrations of O_3_ are usually higher on sunny days with lower PMs concentrations, so people are more likely to misjudge the air quality and ignore its potential risks. And currently the most efforts to improve air quality focus on controlling the concentrations of PMs. Therefore the ambient O_3_ also deserved great attention in both environment protection and in public education.

There are also some deficiencies in this study. First, although over 26,000 CEVD death cases were included, the outcomes of the multi-center studies can be more convincible. Second, the findings in the exposure-response curves cannot be completely explained by the current evidences. Therefore, more studies on the gender-based differences in the vulnerability to ambient air pollution are needed.

## 5 Conclusions

Women were generally more vulnerable to short-term air pollution exposure than men in Shenyang. Ozone can induce excess daily CEVD mortality, which deserves more attention in public health education.

## Additional File

The additional file for this article can be found as follows:

10.5334/gh.849.s1 Supplementary file. Figure S1.
